# High Rates of Advanced Chronic Liver Disease in Patients With Chronic Hepatitis D Virus Infection in Uzbekistan

**DOI:** 10.1111/liv.70799

**Published:** 2026-07-16

**Authors:** Malika E. Khodjaeva, Aziza S. Khikmatullaeva, Nargiz S. Ibadullaeva, Gulnara K. Khudaykulova, Munisa M. Ilyasova, Kamila U. Eshbayeva, Dildorakhon N. Abdurakhmanova, Helenie Kefalakes, Petra Huber, Michael J. Gebel, Erkin I. Musabaev, Heiner Wedemeyer, Lisa Sandmann

**Affiliations:** ^1^ Scientific Research Institute of Virology Tashkent Uzbekistan; ^2^ Tashkent Medical State University Tashkent Uzbekistan; ^3^ Department of Gastroenterology, Hepatology, Infectious Diseases and Endocrinology Hannover Medical School Hannover Germany; ^4^ D‐SOLVE Consortium, an EU Horizon Europe‐Funded Project (No 101057917) Hannover Germany; ^5^ Excellence Cluster RESIST, Excellence Initiative Excellence Initiative Hannover Medical School Hannover Germany; ^6^ German Center for Infection Research (DZIF) Hannover/Braunschweig Germany

**Keywords:** Central Asia, CHD, HCC, HDV, hepatitis delta

## Abstract

**Background and Aims:**

Chronic hepatitis B virus (HBV) infection is endemic in Uzbekistan. Previous data showed a high prevalence of anti‐hepatitis D virus (HDV) antibodies in HBsAg‐positive individuals. Limited data are available on disease severity, treatment availability and hepatocellular carcinoma (HCC) prevalence in patients with chronic HDV infection in Uzbekistan.

**Methods:**

The Scientific Research Institute of Virology (SRIoV) is the leading medical and scientific reference centre for viral hepatitis in Uzbekistan. In this retrospective study, epidemiological, clinical and laboratory data were collected and analysed from all HBsAg‐positive patients presenting to the SRIoV between June 2023 and May 2024. Data from HDV‐coinfected and HBV‐monoinfected patients were compared.

**Results:**

Data of 1393 individual patients were included. HDV coinfection was more prevalent than HBV‐monoinfection (73.9% [*n* = 1030/1393]) vs. 26.1% [*n* = 363/1393]. HDV‐infected patients were significantly younger (41.9 vs. 45.4, *p* < 0.001) and showed higher rates of cirrhosis (78.4% vs. 43.9%, *p* < 0.001) with the majority of patients being classified as Child–Pugh score B (57.8%). Only a minority of HDV‐coinfected patients has ever received anti‐HDV‐directed treatment (0.4%, *n* = 4/1030). Consecutively, HDV RNA was detectable in 78.9% (812/1023) of patients. Quantitative HDV RNA was independently associated with ALT elevation > upper limit of normal. The HCC detection rate was low in both patient groups due to insufficient screening measures (HDV: *n* = 7/1030, HBV: *n* = 11/363).

**Conclusions:**

In this retrospective cohort of 1393 individual patients, HDV coinfection exceeds HBV monoinfection in prevalence and disease severity. Most HDV‐infected patients present with advanced liver disease and currently lack access to HDV‐directed therapy.

## Key Points

- In this retrospective cohort of 1393 individual patients, chronic HDV coinfection in Uzbekistan exceeds HBV monoinfection in prevalence and disease severity.
- Higher HDV RNA is independently associated with ALT elevation above the upper limit of normal.
- Currently, only a minority of patients receive HDV‐directed treatment in Uzbekistan.

## Lay Summary

In this large retrospective cohort from Uzbekistan, HDV coinfection was more prevalent than HBV mono infection and was associated with significantly higher rates of cirrhosis and advanced liver disease. The majority of HDV‐infected patients had detectable HDV RNA and almost no prior exposure to HDV‐directed therapy, indicating a substantial unmet therapeutic need. Improved access to antiviral treatment and strengthened HCC surveillance are urgently required to address the high burden of HDV‐associated liver disease.

**Abbreviations**
AFP: alpha‐fetoprotein
ALT: alanine aminotransferase
AST: aspartate aminotransferase
HBV: hepatitis B virus
HCC: hepatocellular carcinoma
HCV: hepatitis C virus
HDV: hepatitis D virus
HIV: human immunodeficiency virus
IFN: interferon
IQR: interquartile range
NA: nucleos(t)ide analogue
PCR: polymerase chain reaction
SRIoV: Scientific Research Institute of Virology
ULN: upper limit of normal

## 1 Introduction

Prevalence of chronic hepatitis D virus (HDV) infection varies globally and regions with high endemicity have been reported in Central Asia [1]. HDV coinfection is characterised by faster disease progression, a higher risk of developing cirrhosis and hepatocellular carcinoma (HCC), and often a worse prognosis compared to HBV monoinfection [2, 3, 4]. Since HDV infection requires simultaneous or pre‐existing hepatitis B virus (HBV) infection [5], countries with high prevalence of HBV infection may also have a high prevalence of HDV coinfection. Despite its global prevalence and severity, HDV remains underestimated in clinical practice. This is due to both limited access to diagnostics and the lack of effective and affordable therapy.

HBV infection is highly prevalent in Uzbekistan [6]. In contrast, data on the prevalence and clinical impact of HDV coinfection remain limited. A retrospective study reported a substantial proportion of anti‐HDV positivity among HBsAg‐positive patients with advanced liver disease [7]. However, detailed data on virological parameters and disease severity, including liver‐related complications, were only available for a small subset of patients. To date, no studies have systematically evaluated treatment uptake or the prevalence of HCC in HDV‐coinfected patients in Uzbekistan.

This retrospective study aims to provide a comprehensive assessment of HDV prevalence, disease severity and treatment uptake at the tertiary hepatology centre in Uzbekistan, the Scientific Research Institute of Virology (SRIoV). As the national referral centre for viral hepatitis, the SRIoV receives patients with viral liver disease from all regions of the country. This centralised referral structure enables a nationwide perspective on HDV epidemiology, encompassing prevalence, clinical characteristics, complication profiles and current treatment practices.

## 2 Patients and Methods

### 2.1 Study Design and Data Collection

This retrospective single‐centre study included all consecutive HDV‐infected patients presenting to the SRIoV from June 2023 to May 2024. All adult patients with positive HBsAg, positive anti‐HDV or HDV RNA and available clinical and laboratory data were included. HBV‐infected patients without HDV coinfection were included for comparison. Inclusion criteria were confirmed chronic HBV infection, absence of serological or molecular markers of HDV infection, age ≥ 18 years and availability of clinical and laboratory data. Patients with acute hepatitis or history of liver transplantation were excluded from both groups.

Clinical, epidemiological and laboratory data were extracted from patients' medical records. The diagnosis of liver cirrhosis was based on a combination of clinical, laboratory and imaging data and was retrieved from medical charts. If available, reports from ultrasound, transient elastography (Fibroscan [Echosens]) and computed tomography (CT) were included. An overview of data availability is displayed in Table S1.

### 2.2 Laboratory and Virological Methods

Antibodies to HCV and HBsAg in serum were determined using ELISA‐ANTI‐HCV and DS‐ELISA‐HBsAg kits (Diagnostic Systems, Nizhny Novgorod). Quantitative and qualitative determination of HBV DNA, HDV RNA and HCV RNA was performed by PCR using AmpliSense HBV‐FL, AmpliSense HDV‐FL and AmpliSense HCV‐FL kits (Central Research Institute of Epidemiology, Russia), respectively. Biochemical and haematological parameters were analysed based on standard laboratory tests.

### 2.3 Statistical Analysis

Statistical analyses were performed using SPSS statistics version 28 (IBM Corp. Released 2021. IBM SPSS Statistics for Windows, Version 28.0. Armonk, NY: IBM Corp) and GraphPad Prism version 10.2.1 for Windows (GraphPad Software, San Diego, California USA). Students' *t*‐test and Mann–Whitney *U* test were used for comparison of parametric and non‐parametric continuous parameters. Pearson's chi‐square test and Fisher's exact test were used to analyse categorical variables. Multivariable linear regression with backward exclusion was used for multivariable analyses. Continuous variables are presented as median and interquartile range (IQR), categorical data as numbers with percentages (valid percentages).

## 3 Results

### 3.1 High Prevalence of HDV Coinfection Compared to HBV Monoinfection

During the 1‐year study period, 1393 consecutive patients with HBV mono‐ or HDV coinfection presented to the SRIoV. The majority of patients were male (53.8%) and at a median age of 42.5 (IQR 37.3–50) years (Table 1). The median time until the presentation at the tertiary centre was 4.4 (IQR 1.7–7.4) years after first diagnosis of viral hepatitis. HCV coinfection was rare and only present in 2.2% of all patients, and none of the patients showed HIV coinfection. HCC was detected in 18 cases (15.4%); however, only a minority of patients received the appropriate and recommended HCC screening by ultrasound (117/1393 [8.4%]) (Table S1). The prevalence of HDV coinfection (73.9%) was higher compared to HBV monoinfection (26.1%). Patients with HDV coinfection were significantly younger (*p* < 0.001) and more likely to show detectable HBV DNA (*p* < 0.001), while having significantly lower quantitative HBV DNA levels (*p* = 0.035). The use of nucleos(t)ide analogues (NA) was higher in patients with HDV coinfection. Prevalence of cirrhosis was significantly higher in HDV‐coinfected patients (78.4%) compared to HBV‐monoinfected patients (43.9%, *p* < 0.001). Further characteristics are depicted in Table 1.

**TABLE 1:** Comparison of patients with HBV monoinfection and HDV coinfection.

Variable	Total cohort (*n* = 1393)	HDV infection (*n* = 1030)	HBV monoinfection (*n* = 363)	*p*
Age (years)	42.5 (37.3–50)	41.9 (37.3–48.7)	45.4 (37.5–56.8)	< 0.001
Sex, male	749 (53.8)	532 (51.7)	217 (59.8)	0.008
HDV RNA positive	813 (58.7)	813 (79.5)	—	
HDV RNA (copies/mL)^a^	130 000 (11 000–720 000)	130 000 (11 000–720 000)	—	
HBV DNA positive	1309 (94)	1006 (97.7)	303 (83.7)	< 0.001
HBV DNA (IU/mL)^b^	500 (78.8–5125)	305 (70.8–2025)	665 (112.8–12 750)	0.035
HCV coinfection	30 (2.2)	9 (0.9)	21 (5.8)	< 0.001
Cirrhosis	967 (69.5)	808 (78.4)	159 (43.9)	< 0.001
Prior decompensation^c^	106 (11)	88 (10.9)	18 (11.3)	0.910
Hepatocellular carcinoma^d^	18 (15.4)	7 (7.1)	11 (61.1)	0.002
NA therapy	940 (67.5)	724 (70.4)	216 (59.5)	< 0.001
Years since diagnosis	4.4 (1.7–7.4)	4.4 (1.7–7.1)	4.8 (1.5–8.6)	0.162
Diagnosis < 12 months	188 (13.5)	127 (12.3)	61 (16.8)	0.032

*Note:* Continuous parameters are depicted as median with interquartile range, categorical variables as number with percentages. Mann–Whitney *U* test, chi‐square or Fisher's exact test were used for group comparison.

Abbreviations: ALT, alanine aminotransferase; AST, aspartate aminotransferase; HBV, hepatitis B virus; HCC, hepatocellular carcinoma; HCV, hepatitis C virus; HDV, hepatitis D virus; IFN, interferon; NA, nucleos(t)ide analogue.

^a^ HDV RNA positive samples only (*n* = 812).

^b^ HBV DNA positive samples only (*n* = 1309).

^c^ Patients with cirrhosis only (*n* = 967 [HDV: *n* = 808, HBV: *n* = 159]).

^d^ Patients with HCC screening only (*n* = 117 [HDV: *n* = 99, HBV: *n* = 18]).

In multivariable analysis, younger age, presence of cirrhosis and absence of HCV coinfection were independently associated with HDV coinfection (Table S2).

### 3.2 High Prevalence of Advanced Liver Disease in Patients With HDV Coinfection

The overall number of individual HDV‐coinfected patients presenting to the SRIoV was high (*n* = 1030). Female (48.3%) and male (51.7%) patients were evenly distributed within the cohort, and the majority of patients (78.9%) had detectable HDV RNA (Table 2). The median time since initial diagnosis was 4.4 years (IQR 1.7–7.1). HDV coinfection was diagnosed in the same year as presentation to the SRIoV in only 12.3% (127/1030) of patients. Cirrhosis was present in 78.4% (808/1030) of patients with 30.8%, 57.8% and 11.5% of patients being classified as Child–Pugh score A, B and C, respectively. Consecutively, the subgroup of patients with cirrhosis showed significantly lower median levels of albumin and platelets, while median levels of bilirubin were significantly higher compared to HDV‐coinfected patients without cirrhosis (Table 2). Patients with cirrhosis were significantly older (*p* < 0.001) and more likely to be male (*p* = 0.026). Signs of portal hypertension defined as presence of ascites or platelet counts < 50 × 1000/μL and spleen size > 135 mm were present in 76.9% (621/808) of patients with cirrhosis. Interestingly, ALT levels were only slightly elevated (38.4 U/L [IQR 32.8–51.2]) in the total HDV‐coinfected cohort and comparable between patients with and without cirrhosis (*p* = 0.075). Furthermore, HDV RNA detectability rates (79.6% vs. 79%, *p* = 0.851) as well as HDV RNA levels did not differ between cirrhotic and non‐cirrhotic HDV‐coinfected patients (140 000 [IQR 11 150–670 000] vs. 113 500 copies/mL [IQR 10 000–1 365 000], *p* = 0.767). The majority of HDV‐coinfected patients received NA treatment (70.4%), while only a minority of patients had ever received treatment with interferon (0.4%). No significant differences in treatment were observed between patients with and without cirrhosis.

**TABLE 2:** Clinical and biochemical characteristics of patients with HDV coinfection with and without cirrhosis.

Variable	HDV coinfection (*n* = 1030)	Cirrhosis (*n* = 808)	No cirrhosis (*n* = 222)	*p*
Age (years)	41.9 (37.3–48.7)	42.4 (37.8–49.4)	40.5 (36.2–45.8)	< 0.001
Sex, male	532 (51.7)	432 (53.5)	100 (45.1)	0.026
HDV RNA positive	813 (78.9)	640 (79.6)	173 (79)	0.851
HDV RNA (copies/mL)^a^	130 000 (11 000–720 000)	140 000 (11 150–670 000)	113 500 (10 000–1 365 000)	0.767
HBV DNA positive	1006 (97.7)	789 (97.6)	217 (97.7)	0.931
HBV DNA (IU/mL)^b^	305 (71–2025)	315 (64.8–2650)	225 (73.8–1365)	0.737
HCV coinfection	9 (0.9)	7 (0.9)	2 (0.9)	1.0
ALT (U/L)	38.4 (32.8–51.2)	39.0 (33–51.3)	36.4 (32–50)	0.075
AST (U/L)	32 (30–37)	32.4 (30–37.6)	31.2 (30–35)	0.012
Bilirubin (μmol/L)	17.9 (15.6–39.6)	22.0 (16.3–51.2)	16.0 (13.6–17.2)	< 0.001
Albumin (g/L)	30 (27.6–32.1)	29.4 (27–32)	32.0 (30.0–33.9)	< 0.001
Haemoglobin (g/L)	111 (95–120)	107 (92–118)	117 (110–124)	< 0.001
Platelets (×1000/μL)	120 (81–160)	100 (75–135)	180 (158–213)	< 0.001
Quick	0.86 (0.79–0.90)	0.86 (0.79–0.90)	0.89 (0.86–0.95)	< 0.001
Child–Pugh score				
A		246 (30.8)		
B		462 (57.8)		
C		92 (11.5)		
Spleen size (mm)	152 (120–187)	165 (137–197)	113 (100–117)	< 0.001
FibroScan (kPa)	17 (11–26.6)	22.0 (16.0–30.7)	10.0 (8.0–12.1)	< 0.001
Hepatocellular carcinoma^c^	7 (7.1)	7 (7.8)	0 (0)	1.0
NA therapy	724 (70.4)	579 (71.7)	145 (65.3)	0.063
Previous IFN therapy	4 (0.4)	3 (0.4)	1 (0.5)	1.0
Years since diagnosis	4.4 (1.7–7.1)	4.4 (1.7–7)	4.3 (1.7–7.2)	0.973
Diagnosis < 12 months	127 (12.3)	103 (12.7)	24 (10.8)	0.437

*Note:* Continuous parameters are depicted as median with interquartile range, categorical variables as number with percentages. Mann–Whitney *U* test, chi‐square or Fisher's exact test were used for group comparison.

Abbreviations: ALT, alanine aminotransferase; AST, aspartate aminotransferase; HBV, hepatitis B virus; HCC, hepatocellular carcinoma; HCV, hepatitis C virus; HDV, hepatitis D virus; IFN, interferon; NA, nucleos(t)ide analogue.

^a^ HDV RNA positive samples only (*n* = 812).

^b^ HBV DNA positive samples only (*n* = 1006).

^c^ Patients with HCC screening only (*n* = 99 [cirrhosis: 90; no cirrhosis: 9]).

In multivariable analysis, only older age was independently associated with the diagnosis of cirrhosis in patients with HDV coinfection after adjusting for known parameters associated with cirrhosis (bilirubin, albumin, platelets, quick and spleen size) (Table S3).

### 3.3 Higher HDV RNA Independently Associated With Elevated ALT Levels

The majority of patients with HDV coinfection (58.9%) showed ALT levels above the sex‐specific upper limit of normal (ULN), defined as 42 U/L for men and 32 U/L for women (Table 3). In univariate analysis, patients with ALT > ULN showed higher median levels of HDV RNA (*p* = 0.017), AST (*p* < 0.001) and bilirubin (*p* = 0.024) and lower median haemoglobin levels (*p* = 0.016). Patients with ALT > ULN were more frequently female and less likely to receive NA therapy. No significant differences were detected for age, HBV DNA or prevalence of cirrhosis. ALT levels and frequencies of ALT > ULN were comparable across age groups (Table S4). In multivariable analysis, HDV RNA (aOR 1.209 per log_10_ copies/mL), sex (aOR 0.249 for male), bilirubin (aOR 1.006 per μmol/L) and haemoglobin (aOR 1.013 per g/L) were independently associated with ALT > ULN (Table 4).

**TABLE 3:** Comparison of patients with HDV coinfection and normal or elevated ALT levels above the upper limit of normal.

Variable	ALT > ULN (*n* = 607)	ALT ≤ ULN (*n* = 423)	*p*
Age (years)	42.1 (37.5–49.6)	41.5 (37.2–47.6)	0.191
Sex, male	245 (40.4)	287 (67.8)	< 0.001
HDV RNA positive	466 (77.3)	347 (82.6)	0.041
HDV RNA (copies/mL)^a^	160 000 (15 500–780 000)	100 000 (7300–612 000)	0.017
HBV DNA positive	594 (97.9)	412 (97.4)	0.677
HBV DNA (IU/mL)^b^	255 (66–1450)	400 (72–4025)	0.699
HCV coinfection	4 (0.7)	5 (1.2)	0.500
AST (U/L)	35.4 (31.6–42.3)	30.4 (28.9–32.4)	< 0.001
Bilirubin (μmol/L)	18 (15.9–48)	17.6 (15.1–32.4)	0.024
Albumin (g/L)	29.9 (27.6–32)	30 (27.6–32.4)	0.528
Haemoglobin (g/L)	110 (94–118)	112 (95–122)	0.016
Platelets (×1000/μL)	118 (81–158)	123 (80.1–166)	0.290
Quick	0.86 (0.79–0.90)	0.86 (0.79–0.90)	0.181
Cirrhosis	481 (79.2)	327 (77.3)	0.488
Child–Pugh score			0.913
A	151 (24.9)	100 (23.6)	
B	271 (44.6)	192 (45.4)	
C	55 (9.1)	37 (8.7)	
Spleen size (mm)	149 (120–185)	154 (119–188)	0.568
Portal hypertension	369 (60.8)	262 (61.9)	0.745
FibroScan (kPa)	18 (11.3–27)	16 (10–25.1)	0.294
Hepatocellular carcinoma^c^	4 (8.9)	3 (5.6)	0.699
NA therapy	410 (67.7)	314 (74.2)	0.026
Previous IFN therapy	3 (0.5)	1 (0.2)	0.648
Years since diagnosis	4.5 (1.8–7.1)	4.1 (1.7–6.9)	0.405
Diagnosis < 12 months	74 (12.2)	53 (12.5)	0.923

*Note:* Continuous parameters are depicted as median with interquartile range, categorical variables as number with percentages. Mann–Whitney *U* test, chi‐square or Fisher's exact test were used for group comparison.

Abbreviations: ALT, alanine aminotransferase; AST, aspartate aminotransferase; HBV, hepatitis B virus; HCC, hepatocellular carcinoma; HCV, hepatitis C virus; HDV, hepatitis D virus; IFN, interferon; NA, nucleos(t)ide analogue; ULN, upper limit of normal.

^a^ HDV RNA positive samples only (*n* = 812).

^b^ HBV DNA positive samples only (*n* = 1006).

^c^ Patients with HCC screening only (*n* = 99 [ALT ≤ ULN: 54; ALT > ULN: 45]).

**TABLE 4:** Multivariable analysis of parameters associated with ALT > upper limit of normal.

Variable	*p*	Adjusted odds ratio	95% confidence interval
Sex (male)	< 0.001	0.249	0.173–0.356
HDV RNA (per log_10_ copies/mL)	0.002	1.209	1.074–1.361
Haemoglobin (per g/L)	0.021	1.013	1.002–1.024
NA therapy (yes)	0.696	0.929	0.641–1.345
Bilirubin (per μmol/L)	0.010	1.006	1.002–1.011

*Note:* Binary logistic regression with backward exclusion was performed. AST was not included in the multivariable model due to significant correlation to ALT.

Abbreviations: ALT, alanine aminotransferase; AST, aspartate aminotransferase; HBV, hepatitis B virus; HDV, hepatitis D virus; NA, nucleos(t)ide analogue.

### 3.4 Low HCC Screening Rates During the Study Period

Despite high ultrasound report availability, screening rates for HCC were low (8.4%, 117/1393) (Table 1). In HBV‐infected patients, HCC was diagnosed in 61.1% (11/18) of patients who received HCC screening by ultrasound. However, only 7.5% (12/159) of all HBV‐monoinfected patients with cirrhosis received HCC screening. In HDV coinfection, HCC screening rates were comparably low. Only 11.1% (90/808) of HDV‐coinfected patients with cirrhosis received HCC screening, leading to an HCC detection rate of 7% (7/90) in these patients (Table 2). HCC incidence was comparable between patients with and without ALT elevation (Table 3). However, due to the overall low detection rate, these results have to be taken with caution.

### 3.5 Geographical Heterogeneity Among Patients With HDV Coinfection and Cirrhosis in Uzbekistan

The region of residence in Uzbekistan was assessed during data collection. Most HDV‐coinfected patients were referred from Surkhandarya (19.5%), Tashkent (17.9%) and Bukhara (17.8%) region. Only few patients were referred from Khorezm (1.3%) or Jizzakh (0.7%) region or the Republic of Karakalpakstan (0.3%). Cirrhosis prevalence rates differed according to region of origin, with Jizzakh (85.7%), Namangan (85.4%) and Surkhandarya (84.7%) region showing the highest rates of cirrhosis, while Navoi (52.6%) and Syrdarya (54.1%) region showed the lowest prevalence of cirrhosis. However, each of these regions, except for the Surkhandarya region, contributed to less than 10% of the patients of the cohort (Table S5).

## 4 Discussion

The study provides a comprehensive analysis of clinical characteristics of patients with HBV mono‐ and HDV coinfection presenting to the national referral centre for viral hepatitis in Uzbekistan. The study demonstrates that the prevalence of HDV coinfection exceeded HBV monoinfection in the study population. Despite their significantly younger age, rates of cirrhosis were significantly higher in patients with HDV coinfection. Within the subgroup of HDV‐coinfected patients, older age was independently associated with the diagnosis of cirrhosis, while no differences were detected for HDV viremia. Irrespective of age and stage of liver disease, HDV RNA levels were independently associated with ALT elevation > ULN. Only few HCC cases were detected, demonstrating the overall low rate of HCC screening measures during the study period.

Recent results of a nationwide screening programme demonstrated HBV prevalence rates between 2.14% and 3.96% in Uzbekistan depending on region of residency and age. Significantly lower rates were present among individuals born after 2000, the year of full coverage of HBV vaccination and highest rates for those ageing between 41 and 50 years [8]. This is in line with the results from our study showing a median age of 42.5 years of the total HBsAg‐positive cohort. So far, no nationwide screening programme for the detection of HDV coinfection has been conducted in Uzbekistan. Previously published data analysing anti‐HDV prevalence in HBsAg‐positive patients with cirrhosis presenting to the SRIoV from 2016 to 2018 demonstrated a high anti‐HDV prevalence ranging from 76.5% to 84% [7]. Clinical data of 138 HDV‐coinfected patients with cirrhosis were included and showed a Child–Pugh score distribution of 38%, 50% and 12% for A, B and C, respectively. The present study provides a more detailed analysis of clinical characteristics of all HBsAg‐positive patients presenting to the national referral centre within one year. HDV coinfection rates were confirmed (73.9%) as was the high prevalence of cirrhosis (78.4%) in HDV‐coinfected patients. Several studies have already attributed HDV coinfection to a higher risk for the development of advanced liver disease [4, 9, 10] and HCC [3, 11]. Our results are in line with these previously published studies, which were conducted in countries outside Central Asia. A recent study from Mongolia comparing 2382 treatment‐naïve HBsAg‐positive adults with HDV coinfection to 1553 HBV‐monoinfected patients confirmed the overall higher rate of cirrhosis in HDV coinfection [12]. Importantly, ALT elevations were more prevalent in younger HDV‐coinfected individuals, which was associated with higher rates of cirrhosis compared to age‐matched HBV‐monoinfected patients. In the present study, ALT elevation was not associated with age and median ALT levels were comparable between patients at an age of 18–30, 30–40, 40–50 or > 50 years, respectively. Despite the overall only slightly elevated median ALT levels (38.4 U/L [IQR 32.8–51.2 U/L]), a significant proportion of patients (58.9%) showed ALT values > ULN when stratifying according to sex‐specific ULN. Importantly, HDV RNA levels were independently associated with ALT > ULN in HDV‐coinfected patients underlining the relevance of viremia in patients with HDV coinfection [13].

HDV genotyping has gained increasing attention because of its potential association with disease progression, clinical outcomes and treatment response. Recent studies have demonstrated that HDV genotype 1 exhibits substantially greater genetic diversity than previously recognised, leading to the identification of several novel subgenotypes [14]. However, data on HDV genotype distribution in Uzbekistan remain scarce. Due to limited access to sequencing technologies and laboratory resources, HDV genotyping has not yet been routinely implemented in the country. Efforts to establish HDV genotyping at the SRIoV are currently underway, and future studies investigating the genotype distribution and molecular epidemiology of HDV in Uzbekistan are expected to provide valuable insights into the regional characteristics of HDV infection.

Only a minority of HDV‐coinfected patients received interferon‐based treatment (0.4%), and no access to other HDV‐directed antiviral therapies or clinical trials for chronic HDV infection is currently available in Uzbekistan. The high proportion of patients with decompensated liver disease (69.3%), together with the lack of coverage by health insurance or government programmes, may explain the low interferon treatment rate. In contrast, a substantial proportion of patients received NA therapy, demonstrating the general feasibility of antiviral treatment. Notably, HDV‐coinfected patients were significantly more likely to receive NA therapy than HBV‐monoinfected patients. This may partly be explained by the higher prevalence of cirrhosis among HDV‐coinfected patients, thereby fulfilling treatment criteria in the presence of detectable HBV [15, 16], as well as the relatively low HBV DNA levels observed in HBV‐monoinfected patients (median 665 IU/mL [IQR 112.8–12 750 IU/mL]). However, only 63% of HBV DNA–positive HBV‐monoinfected patients underwent quantitative HBV DNA testing; therefore, these findings should be interpreted with caution. The significantly younger age and higher prevalence of advanced liver disease (cirrhosis with portal hypertension) among patients with HDV coinfection underscore the need to improve treatment access in this population.

Several studies have demonstrated an increased HCC‐risk in patients with HDV coinfection [3, 11, 17]. In the present study, HCC was diagnosed in only seven HDV‐coinfected patients, accounting for 0.7% of the entire HDV cohort and 0.9% of the HDV‐coinfected patients with cirrhosis. This rate is lower than the expected risk of HCC development in patients with HDV‐associated cirrhosis described in epidemiological and clinical studies [18]. The most likely explanation is the limited availability and irregularity of HCC screening, as early detection diagnostics are currently not part of the standard medical care in Uzbekistan.

This study has important limitations. First, the retrospective design precludes the establishment of causal relationships between individual factors and the course of HDV infection or the development of complications. In addition, the absence of longitudinal follow‐up data limits the ability to assess patient trajectories over time. Second, the study includes only patients presenting to the SRIoV, introducing potential selection bias. It has been shown that patients presenting to tertiary centres have more advanced liver disease compared to those presenting to secondary centres [2]. Whether this is also true for the HDV‐coinfected population in Uzbekistan is unclear. However, as the SRIoV is the national reference centre for viral hepatitis and chronic liver diseases, receiving referrals from across the country, the cohort likely reflects the broader population of HBV‐ and HDV‐infected patients accessing the healthcare system in Uzbekistan. Third, incomplete availability of certain laboratory and clinical parameters may have led to an underestimation of specific outcomes, particularly HCC. Moreover, the lack of standardised and regular oncological screening further limits accurate assessment of the true prevalence of HCC within the cohort. Finally, data on antiviral therapy, including interferon treatment, were limited, precluding a detailed evaluation of the effectiveness of different therapeutic strategies in patients with HDV infection. Despite these limitations, the large sample size and the comprehensive analysis of clinical, laboratory and virological parameters, including quantitative HDV RNA levels, from this Central Asian region represent a unique strength of the present study.

In summary, this study highlights the high prevalence and, given its substantial morbidity, the significant clinical burden of HDV coinfection in Uzbekistan. Limited access to HDV‐specific therapies, together with the late stage at which patients present for medical care, represent key areas for intervention to improve clinical outcomes. The results emphasise the need to expand screening programmes, diagnostics and regular clinical monitoring of patients with HDV infection in regions with high endemicity.

## Author Contributions

The study was designed and coordinated by M.E.K., P.H., E.I.M., H.W. and L.S. Patients were identified and data collected by A.S.K., N.S.I., G.K.K., M.M.I., K.U.E. and D.N.A. Data analysis, data interpretation and statistics were performed by M.E.K., H.K. and L.S. Data interpretation was done by M.E.K., H.K., M.J.G., E.I.M., H.W. and L.S. Drafting of the manuscript was done by M.E.K. and L.S. and critical revision of the manuscript was performed by all authors. All authors approved the final manuscript.

## Funding

The project was supported by the hospital partnership programme of Deutsche Gesellschaft für Internationale Zusammenarbeit (GIZ) (No. 17.2170.3‐002.22).

## Ethics Statement

This study was conducted in accordance with current guidelines and regulations and the principles of the Declaration of Helsinki. Ethical approval for the study was granted by the Ethics Committee of the Ministry of Health of the Republic of Uzbekistan under protocol number 5/12‐1893 dated 1 June 2024.

## Consent

The requirement for written informed consent was waived, as the study involved the analysis of retrospective routine clinical data obtained from medical records.

## Conflicts of Interest

H.W. served as a speaker and/or advisory board member for Abbott, Alfasigma GmbH, Aligos Therapeutics Inc., Atea Pharmaceuticals Inc., BioMarin Pharmaceuticals Inc., Biotest AG, Bluejay Therapeutics, Bristol‐Myers‐Squibb, CSL Behring, Dr. Falk Pharma GmbH, EIT Pharma Inc., F. Hoffmann/La Roche Ltd., Gilead Science, GlaxoSmithKline Services Unlimited, IntergerBio Inc., IPSEN Pharma, Lilly Deutschland GmbH, Mirum Pharmaceuticals Germany GmbH, Orphalan GmbH, Primmune Therapeutics Inc., Ribocure Pharmaceuticals, SANOFI Aventis Deutschland GmbH, Takeda Pharma, Verein Falk Foundation e.V. and Vir Biotechnology Inc. H.W. works as an Investigator in clinical trials by BioMarin Pharmaceuticals Inc., Bluejay Therapeutics, Dr. Falk Pharma GmbH, Gilead Science, GlaxoSmithKline Services Unlimited, IPSEN Pharma, Lilly Deutschland GmbH, Mirum Pharmaceuticals Germany GmbH, Takeda Pharma and Vir Biotechnology Inc. and received research grants from Biotest AG and Gilead Science. H.K. received research grants from Gilead. L.S. served as a speaker and/or advisory board member for Roche and Gilead and received travel support by Abbvie, Gilead, Falk Pharma and research grants from Gilead. The other authors declare no conflicts of interest.

## Supporting information


**Table S1:** Overview of available and missing clinical, laboratory and virological data (*n* = 1030).
**Table S2:** Multivariable analysis of parameters associated with HDV coinfection. Binary logistic regression with backward exclusion was performed.
**Table S3:** Multivariable analysis of parameters associated with HDV coinfection and cirrhosis. Binary logistic regression with backward exclusion was performed. Due to significant correlation of bilirubin, albumin, platelets, quick and spleen size, different models were analysed.
**Table S4:** Number and proportion of patients with ALT > ULN (A) and median ALT levels (B) stratified by age. Continuous parameters are depicted as median with interquartile range, categorical variables as number with percentages.
**Table S5:** Geographical distribution of HDV coinfected patients and frequency of cirrhosis depending on the region of residence.

## Data Availability

The data that support the findings of this study are available from the corresponding author upon reasonable request.

## References

[1] A. J. Stockdale , B. Kreuels , M. Y. R. Henrion , et al., “The Global Prevalence of Hepatitis D Virus Infection: Systematic Review and Meta‐Analysis,” Journal of Hepatology 73, no. 3 (2020): 523–532, 10.1016/j.jhep.2020.04.008. 32335166 PMC7438974

[2] H. Kamal , G. Westman , K. Falconer , et al., “Long‐Term Study of Hepatitis Delta Virus Infection at Secondary Care Centers: The Impact of Viremia on Liver‐Related Outcomes,” Hepatology 72, no. 4 (2020): 1177–1190, 10.1002/hep.31214. 32145073

[3] H. Kamal , R. Fornes , J. Simin , et al., “Risk of Hepatocellular Carcinoma in Hepatitis B and D Virus Co‐Infected Patients: A Systematic Review and Meta‐Analysis of Longitudinal Studies,” Journal of Viral Hepatitis 28, no. 10 (2021): 1431–1442, 10.1111/jvh.13577. 34291520

[4] A. Wranke , B. Heidrich , K. Deterding , et al., “Clinical Long‐Term Outcome of Hepatitis D Compared to Hepatitis B Monoinfection,” Hepatology International 17, no. 6 (2023): 1359–1367, 10.1007/s12072-023-10575-0. 37789170 PMC10661878

[5] M. Rizzetto , M. G. Canese , S. Arico , et al., “Immunofluorescence Detection of New Antigen‐Antibody System (Delta/Anti‐Delta) Associated to Hepatitis B Virus in Liver and in Serum of HBsAg Carriers,” Gut 18, no. 12 (1977): 997–1003, 10.1136/gut.18.12.997. 75123 PMC1411847

[6] U. Y. Ismoilov , E. I. Musabaev , A. S. Khikmatullaeva , et al., “Hepatitis B and C Landscape in Uzbekistan: Epidemiological Patterns Revealed in a Study of 1 040 000 People,” European Journal of Public Health 35, no. 5 (2025): 1014–1019, 10.1093/eurpub/ckaf136. 40843480 PMC12529272

[7] M. Khodjaeva , N. Ibadullaeva , A. Khikmatullaeva , et al., “The Medical Impact of Hepatitis D Virus Infection in Uzbekistan,” Liver International 39, no. 11 (2019): 2077–2081, 10.1111/liv.14243. 31505080

[8] E. Musabaev , C. Estes , S. Sadirova , et al., “Viral Hepatitis Elimination Challenges in Low‐ and Middle‐Income Countries‐Uzbekistan Hepatitis Elimination Program (UHEP),” Liver International 43, no. 4 (2023): 773–784, 10.1111/liv.15514. 36606729

[9] Z. Miao , S. Zhang , X. Ou , et al., “Estimating the Global Prevalence, Disease Progression, and Clinical Outcome of Hepatitis Delta Virus Infection,” Journal of Infectious Diseases 221, no. 10 (2020): 1677–1687, 10.1093/infdis/jiz633. 31778167 PMC7184909

[10] B. V. John , D. Bastaich , M. M. Amoli , et al., “Association of HDV Infection and HCC, Hepatic Decompensation, and All‐Cause and Liver‐Related Death in a National Cohort,” Hepatology 81, no. 6 (2025): 1822–1835, 10.1097/HEP.0000000000001092. 39255517

[11] D. Alfaiate , S. Clement , D. Gomes , N. Goossens , and F. Negro , “Chronic Hepatitis D and Hepatocellular Carcinoma: A Systematic Review and Meta‐Analysis of Observational Studies,” Journal of Hepatology 73, no. 3 (2020): 533–539, 10.1016/j.jhep.2020.02.030. 32151618

[12] H. Kamal , G. Jargalsaikhan , S. Enkhtaivan , et al., “Prevalence and Risk Factors of Elevated Alanine Aminotransferase (ALT) in 2382 Treatment‐Naive HBV/HDV co‐Infected Patients,” Liver International 46, no. 4 (2026): e70559, 10.1111/liv.70559. 41749331 PMC12946601

[13] R. G. Gish , R. J. Wong , G. L. Di Tanna , et al., “Association of Hepatitis Delta Virus With Liver Morbidity and Mortality: A Systematic Literature Review and Meta‐Analysis,” Hepatology 79, no. 5 (2024): 1129–1140, 10.1097/HEP.0000000000000642. 37870278 PMC11019996

[14] S. Manhas , S. Chang , R. Martin , et al., “Classification and Sequencing of Hepatitis D Virus From a Large Cohort of Chronically Infected Individuals Paired With Co‐Infecting Hepatitis B Virus Sequencing: A Genomic Characterisation Study,” Lancet Microbe 7, no. 5 (2026): 101328, 10.1016/j.lanmic.2025.101328. 41969017

[15] European Association for the Study of the Liver (EASL) , “EASL Clinical Practice Guidelines on Hepatitis Delta Virus,” Journal of Hepatology 79, no. 2 (2023): 433–460, 10.1016/j.jhep.2023.05.001. 37364791

[16] European Association for the Study of the Liver (EASL) , “EASL Clinical Practice Guidelines on the Management of Hepatitis B Virus Infection,” Journal of Hepatology 83 (2025): 502–583, 10.1016/j.jhep.2025.03.018. 40348683

[17] R. Romeo , E. Del Ninno , M. Rumi , et al., “A 28‐Year Study of the Course of Hepatitis Delta Infection: A Risk Factor for Cirrhosis and Hepatocellular Carcinoma,” Gastroenterology 136, no. 5 (2009): 1629–1638, 10.1053/j.gastro.2009.01.052. 19208358

[18] G. Fattovich , G. Giustina , E. Christensen , et al., “Influence of Hepatitis Delta Virus Infection on Morbidity and Mortality in Compensated Cirrhosis Type B. The European Concerted Action on Viral Hepatitis (Eurohep),” Gut 46, no. 3 (2000): 420–426, 10.1136/gut.46.3.420. 10673308 PMC1727859

