# Supplementary material for: High Rates of Advanced Chronic Liver Disease in Patients With Chronic Hepatitis D Virus Infection in Uzbekistan
Source: Liver Int. 2026 Jul 16;46(8):e70799. doi: 10.1111/liv.70799 (PMC13376201; doi:10.1111/liv.70799)
Supplement: Supplementary file 1 — Table S1: Overview of available and missing clinical, laboratory and virological data (n = 1030). Table S2: Multivariable analysis of parameters associated with HDV coinfection. Binary logistic regression with backward exclusion was performed. Table S3: Multivariable analysis of parameters associated with HDV coinfection and cirrhosis. Binary logistic regression with backward exclusion was performed. Due to significant correlation of bilirubin, albumin, platelets, quick and spleen size, different models were analysed. Table S4: Number and proportion of patients with ALT > ULN (A) and median ALT levels (B) stratified by age. Continuous parameters are depicted as median with interquartile range, categorical variables as number with percentages. Table S5: Geographical distribution of HDV coinfected patients and frequency of cirrhosis depending on the region of residence. [file LIV-46-0-s001.docx]

**Supplementary material**

Table of content

[Supplementary table 1. 2](#_Toc225238240)

[Supplementary table 2. 4](#_Toc225238241)

[Supplementary table 3. 5](#_Toc225238242)

[Supplementary table 4. 7](#_Toc225238243)

[Supplementary table 5. 8](#_Toc225238244)

Supplementary table 1.

Overview of available and missing clinical, laboratory, and virological data (n=1030).

Abbreviations: AFP, alfa fetoprotein; ALT, alanine aminotransferase; AST, aspartate aminotransferase; HBV, hepatitis B virus; HCC, hepatocellular carcinoma; HCV, hepatitis C virus; HDV hepatitis D virus, HIV, human immunodeficiency virus; IFN, interferon; NA, nucleos(t)ide analog

| Variable | HDV coinfection (n = 1030) | Missing, n (%) | HBV monoinfection (n = 363) | Missing, n (%) |
| --- | --- | --- | --- | --- |
|  | Available, n (%) |  | Available, n (%) |  |
| Age | 1030 (100) | 0 (0) | 363 (100) | 0 (0) |
| Body weight | 1030 (100) | 0 (0) | 362 (99.7) | 1 (0.3) |
| Sex | 1030 (100) | 0 (0) | 363 (100) | 0 (0) |
| Region | 1030 (100) | 0 (0) | 363 (100) | 0 (0) |
| HDV RNA (qualitative) | 1023 (99.3) | 7 (0.7) | - |  |
| HDV RNA (quantitative) ° | 699 (86) | 114 (14) | - |  |
| HBV DNA (qualitative) | 1030 (100) | 0 (0) | 362 (99.7) | 1 (0.3) |
| HBV DNA (quantitative) ^§^ | 153 (15.2) | 853 (84.8) | 191 (63) | 112 (37) |
| HIV coinfection | 1030 (100) | 0 (0) | 363 (100) | 0 (0) |
| HCV coinfection | 1030 (100) | 0 (0) | 361 (99.4) | 2 (0.6) |
| ALT | 1028 (99.8) | 2 (0.2) | - |  |
| AST | 660 (64.1) | 370 (35.9) | - |  |
| Bilirubin | 1026 (99.6) | 4 (0.4) | - |  |
| Albumin | 952 (92.4) | 78 (7.6) | - |  |
| Hemoglobin | 1024 (99.4) | 6 (0.6) | - |  |
| Platelets | 1023 (99.3) | 7 (0.7) | - |  |
| Quick | 771 (74.9) | 259 (25.1) | - |  |
| Cirrhosis | 1030 (100) | 0 (0) | 362 (99.7) | 1 (0.3) |
| Ascites^#^ | 802 (99.3) | 6 (0.7) |  |  |
| Child-Pugh-Score^#^ | 800 (77.7) | 8 (22.3) |  |  |
| Prior hepatic decompensation^#^ | 794 (98.3) | 14 (1.7) | 158 (99.4) | 1 (0.6) |
| Ultrasound report | 1030 (100) | 0 (0) |  |  |
| Fibroscan | 191 (18.5) | 839 (81.5) |  |  |
| NA therapy | 1029 (99.9) | 1 (0.1) | 363 (100) | 0 (0) |
| IFN therapy | 1030 (100) | 0 (0) |  |  |
| Previous IFN treatment | 1030 (100) | 0 (0) |  |  |
| HCC diagnostic evaluation performed | 99 (9.6) | 931 (90.4) | 18 (5) | 345 (95) |
| Years since diagnosis | 1030 (100) | 0 (0) | 363 (100) | 0 (0) |
| Diagnosis < 12 months | 1030 (100) | 0 (0) | 363 (100) | 0 (0) |

° HDV RNA positive samples only (n = 813)

§ HBV DNA positive samples only (HDV: n = 1006; HBV: n = 303)

# patients with cirrhosis only (HDV: n = 808; HBV: n = 159)

# Supplementary table 2.

Multivariable analysis of parameters associated with HDV coinfection. Binary logistic regression with backward exclusion was performed.

| Variable | p-value | Adjusted odds ratio | 95% confidence interval |
| --- | --- | --- | --- |
| Age (per year) | <0.001 | 0.959 | 0.937-0.982 |
| Sex (male) | 0.136 | 1.484 | 0.883-2.493 |
| HBV DNA (per 1000 IU/mL) | 0.043 | 1 | 0.999-1 |
| HCV coinfection (yes) | 0.003 | 0.044 | 0.005-0.348 |
| Cirrhosis (yes) | <0.001 | 9.933 | 5.886-16.764 |

# Supplementary table 3.

Multivariable analysis of parameters associated with HDV coinfection and cirrhosis. Binary logistic regression with backward exclusion was performed. Due to significant correlation of bilirubin, albumin, platelets, quick, and spleen size, different models were analyzed.

| Variable | p-value | Adjusted odds ratio | 95% confidence interval |
| --- | --- | --- | --- |
| Model A | | | |
| Age (per year) | 0.002 | 1.039 | 1.014-1.065 |
| Sex (male) | 0.477 | 0.864 | 0.578-1.291 |
| AST (per U/L) | 0.842 | 1.002 | 0.986-1.017 |
| Bilirubin (per µmol/L) | <0.001 | 1.061 | 1.037-1.086 |
| Model B | | | |
| Age (per year) | 0.003 | 1.040 | 1.014-1.068 |
| Sex (male) | 0.556 | 0.881 | 0.577-1.344 |
| AST (per U/L) | 0.212 | 1.012 | 0.993-1.031 |
| Albumin (per g/L) | <0.001 | 0.862 | 0.809-0.919 |
| Model C | | | |
| Age (per year) | 0.008 | 1.040 | 1.010-1.071 |
| Sex (male) | 0.479 | 0.837 | 0.511-1.370 |
| AST (per U/L) | 0.377 | 1.008 | 0.990-1.026 |
| Platelets (per 1000/µl) | <0.001 | 0.970 | 0.965-0.975 |
| Model D | | | |
| Age (per year) | 0.010 | 1.048 | 1.011-1.086 |
| Sex (male) | 0.387 | 0.779 | 0.443-1.372 |
| AST (per U/L) | 0.315 | 1.012 | 0.989-1.035 |
| Quick (per 0.1) | <0.001 | 0.518 | 0.358-0.749 |
| Model E | | | |
| Age (per year) | <0.001 | 1.074 | 1.042-1.108 |
| Sex (male) | 0.867 | 0.956 | 0.569-1.609 |
| AST (per U/L) | 0.851 | 1.002 | 0.984-1.019 |
| Spleen size (per mm) | <0.001 | 1.077 | 1.062-1.092 |

# Supplementary table 4.

Number and proportion of patients with ALT > ULN (A) and median ALT levels (B) stratified by age. Continuous parameters are depicted as median with interquartile range, categorical variables as number with percentages.

Abbreviations: ALT: alanine aminotransferase, AST: aspartate aminotransferase, HBV: hepatitis B virus, HDV: hepatitis D virus, NA: nucleos(t)ide analog

**A**

| Age (years) | ALT > ULN (n = 607) | ALT ≤ ULN (n = 423) |
| --- | --- | --- |
| 18-29.9 | 22 (3.6) | 15 (3.5) |
| 30-39.9 | 209 (34.4) | 153 (36.2) |
| 40-49.9 | 235 (38.7) | 181 (42.8) |
| ≥ 50 | 141 (23.2) | 74 (17.5) |

**B**

| Years | 18-29.9 | 30-39.9 | 40-49.9 | ≥ 50 |
| --- | --- | --- | --- | --- |
| ALT U/L | 39.8 (31.3-52) | 39 (32.8-51) | 37.7 (32.7-50.1) | 39.4 (32.9-52) |

# Supplementary table 5.

Geographical distribution of HDV coinfected patients and frequency of cirrhosis depending on the region of residence.

| Region of Uzbekistan | HDV coinfection (n=1030) | Cirrhosis (n=808) | No cirrhosis (n=222) |
| --- | --- | --- | --- |
| Surkhandarya region | 203 (19.7) | 172 (84.7) | 31 (15.3) |
| Tashkent region | 184 (17.9) | 146 (79.3) | 38 (20.7) |
| Bukhara region | 183 (17.8) | 149 (81.4) | 34 (18.6) |
| Tashkent | 113 (11) | 81 (71.7) | 32 (28.3) |
| Samarkand region | 64 (6.2) | 47 (73.4) | 17 (26.6) |
| Andijan region | 63 (6.1) | 53 (84.1) | 10 (15.9) |
| Fergana region | 56 (5.4) | 42 (75) | 14 (25) |
| Namangan region | 48 (4.7) | 41 (85.4) | 7 (14.6) |
| Kashkadarya region | 37 (3.6) | 28 (75.7) | 9 (24.3) |
| Syrdarya region | 37 (3.6) | 20 (54.1) | 17 (45.9) |
| Navoi region | 19 (1.8) | 10 (52.6) | 9 (47.4) |
| Khorezm region | 13 (1.3) | 10 (76.9) | 3 (23.1) |
| Jizzakh region | 7 (0.7) | 6 (85.7) | 1 (14.3) |
| Republic of Karakalpakstan | 3 (0.3) | 3 (100) | 0 (0) |
